# Effects of thrombus migration on endovascular treatment outcomes in patients with ischemic stroke: a systematic review and meta-analysis

**DOI:** 10.1007/s00415-025-13609-9

**Published:** 2026-03-09

**Authors:** Hesham Kelani, Mohamed A. Elzayat, Abdelrahman M. Elettreby, Hend Heikal, Maria Farag, Emily Wen Jing Shuai, Bethany Jordyn Thach, Hamza Khelifa, Emily Carrieri, Gabriela Martin Gonzalez, Vaughn Sherman, Ahmed Abd Elazim, Diana Greene-Chandos, Volodymyr Vulkanov, Moshe Mizrahi, Lisa R. Merlin, David Rosenbaum-Halevi, Priyank Khandelwal

**Affiliations:** 1 Department of Neurology, SUNY Downstate Health Sciences University at One Brooklyn Health, Brooklyn, NY USA; 2 Faculty of Medicine, Mansoura University, Mansoura, Egypt; 3 College of Medicine, SUNY Downstate Health Sciences University, Brooklyn, NY USA; 4 Faculty of Medicine, University of Oran, 1 Ahmed Ben Bella, Oran, Algeria; 5 Touro College of Osteopathic Medicine (TOUROCOM) - Harlem, NY Campus, New York, USA; 6 Department of Neurology, Sanford USD Medical Center, Sioux Falls, SD USA; 7 Department of Neurology, School of Medicine, St. Louis University, SSM Health, Saint Louis, MO USA; 8 Department of Neurology, Rutgers, New Jersey School of Medicine, Newark, NJ USA; 9 Departments of Neurology, Physiology and Pharmacology, SUNY Downstate Health Sciences University, Brooklyn, NY USA

**Keywords:** Acute ischemic stroke, Stroke, Thrombus migration, Intravenous thrombolysis, Mechanical thrombectomy, Meta-analysis

## Abstract

**Background:**

Stroke is the world’s second-leading cause of death, with ischemic events accounting for nearly 9 of 10 cases. Rapid endovascular treatment (EVT) is now standard, yet the benefit of giving intravenous tPA beforehand (“bridging therapy”) remains uncertain. One reason: tPA often shifts the clot distally—thrombus migration (TM)—a phenomenon seen in roughly one-fifth of large-vessel strokes that can complicate the procedure but has also been linked to better outcomes. This study evaluates how TM influences clinical outcomes, aiming to clarify whether tPA adds value in patients who receive EVT.

**Methods:**

A comprehensive literature search was conducted across various databases until April 2025 to identify relevant articles. The quality was assessed using the NOS tool and the analysis was performed using RevMan 5 software. Primary outcomes of interest were favorable functional outcomes (modified Rankin Scale score 0–2) and mortality 90 days after stroke.

**Results:**

Thirteen studies (n = 6,198 patients) were identified fulfilling our research question. Thrombus migration was significantly associated with favourable neurological outcomes (mRS 0–2) at 90 days (OR = 1.43; P = 0.025). TM showed no significant impact on other outcomes, including 90-day mortality (OR = 0.86; P = 0.15), symptomatic intracranial hemorrhage (sICH) (OR = 1.12; P = 0.54), any ICH (OR = 1.25; P = 0.4), NIHSS change at discharge (MD = 0.36; P = 0.18) and successful reperfusion rates (TICI 2b–3) (OR = 0.69; P = 0.0686).

**Conclusions:**

Thrombus migration during mechanical thrombectomy was associated with better 90-day functional outcomes. Although thrombus migration might affect complete revasculrization, it may offer clinical benefits by restoring blood flow to larger brain territories.

**Supplementary Information:**

The online version contains supplementary material available at 10.1007/s00415-025-13609-9.

## Introduction

Stroke is an acute neurological condition characterized by compromised cerebral perfusion and is broadly classified into ischemic and hemorrhagic subtypes. According to the Global Burden of Disease (GBD) 2021, stroke is the second leading cause of death and the third leading cause of mortality and disability combined worldwide [1]. The World Health Organization estimates that approximately 12 million incident strokes occur globally each year, with over half (around 7 million) resulting in death [2]. In the United States, stroke ranks as the fifth leading cause of death and contributes significantly to morbidity and long-term disability among adults aged 65 and older. Every 40 s, someone in the U.S. experiences a stroke, totalling approximately 795,000 events annually [3]. Beyond its clinical toll, stroke imposes a substantial economic burden, with global costs estimated to exceed US$890 billion annually—equivalent to 0.66% of global GDP [2]. Due to its high prevalence and profound societal and clinical impact, stroke remains a major focus of global research.

Ischemic strokes account for 87% of all stroke cases. Approximately 62% of single large-vessel occlusions occur in the middle cerebral artery (MCA) territory, followed by the basilar artery (8.8%), the posterior inferior cerebellar artery (PICA) (7.5%), and the anterior cerebral artery (ACA) (6.6%). The remaining major vessels, including the superior cerebellar artery (SCA) and anterior inferior cerebellar artery (AICA), collectively account for about 2% [4]. The global age-standardized incidence rate of ischemic stroke is projected to rise to 89.32 per 100,000 population by 2030. However, mortality and disability-adjusted life years (DALYs) from ischemic stroke may follow a more favourable trend, with projections showing a decrease in global age-standardized death rates to 18.28 and DALYs to 500.37 per 100,000 population. This improvement is largely attributed to timely recognition and intervention in cases of ischemic stroke [5].

The standard of care for acute ischemic stroke emphasizes rapid reperfusion, typically achieved through intravenous tissue-type plasminogen activator (IV tPA), endovascular treatment (EVT), or a combination of both. However, the role of IV tPA prior to EVT—known as "bridging therapy"—remains a subject of ongoing debate. Several studies suggest that IV tPA combined with EVT improves functional outcomes, reduces mortality, and enhances recanalization rates without significantly increasing the risk of intracerebral haemorrhage compared to EVT alone [6–8]. Conversely, other studies report no significant added benefit of bridging therapy in terms of 90-day functional recovery or disability, suggesting IV tPA may be safely omitted when timely EVT is available due to potential risks and limited efficacy [9–11]. These discrepancies may be explained by heterogeneity in patient characteristics, thrombus location (proximal vs. distal), and thrombus composition, all of which influence procedural success.

Thrombus evolution in acute ischemic stroke—encompassing thrombus migration, growth, and resolution—is a well-documented phenomenon [12, 13]. Thrombus migration (TM), defined as a downstream movement of the thrombus observed between initial angiographic imaging (often Computed Tomography Angiography) and subsequent imaging (often Digital Subtraction Angiography), is of particular interest [12, 14]. A large study using the MR CLEAN registry found that 22% of patients experienced thrombus migration, 6% had thrombus growth, and 3% showed thrombus resolution [14]. Ongoing debate surrounds the factors contributing to thrombus migration and how this phenomenon impacts EVT success and patient outcomes. TM appears to be more common among patients receiving IV tPA [15–17]. Despite potential technical challenges posed by IVT-associated thrombus migration, multiple studies have paradoxically reported that TM is associated with better functional outcomes [13, 14, 18, 19]. These findings suggest that thrombus migration may play a more complex and possibly beneficial role in stroke management than previously thought.

This systematic review and meta-analysis aims to evaluate the impact of thrombus migration on EVT outcomes in acute ischemic stroke. Specifically, we evaluated its influence on recanalization rates, functional outcomes as measured by the modified Rankin Scale (mRS), and mortality. Clarifying the role of TM in this context may inform clinical decision-making regarding the use of IV tPA in patients for whom rapid EVT is available and indicated.

## Methods

### Protocol

This systematic review was executed in compliance with the PRISMA statement standards [22] and the directives outlined in the Cochrane Handbook of Systematic Reviews and Meta-Analyses (version 5.1.0) [12]. Before conducting this systematic review, established methodologies and refined criteria were developed, and the protocol was prospectively registered with PROSPERO (ID: CRD420251125424).

### Eligibility criteria

#### Inclusion criteria

The following selection criteria were used to identify published articles to be included in this meta-analysis:- *Population*: Patients with ischemic stroke treated with endovascular treatment.—*Exposure*: Patients in whom the thrombus had migrated.—*Comparator*: Patients in whom the thrombus had not migrated.—*Outcomes*: Safety or efficacy outcomes. Safety outcomes included mortality 90 days after stroke, symptomatic intracranial haemorrhage (sICH) and any intracranial haemorrhage, while efficacy outcomes included successful recanalization rate (defined as Thrombolysis in Cerebral Infarction (TICI) score ≥ 2b), favourable functional outcomes (defined as mRS 0–2) at 90 days.

*Study design:* Observational studies.

### Exclusion criteria

Studies that met any of the following criteria were excluded: 1) other study designs, such as reviews, comments, and case series; 2) studies conducted on non-human subjects, such as animal models; and 3) studies conducted on patients diagnosed with hemorrhagic stroke or who had secondary embolism, not thrombus migration.

### Information sources and search strategy

A comprehensive literature search was conducted across various databases (including PubMed, Scopus, Web of Science, and Cochrane database) using a tailored search strategy (Supplementary file 1) with some modifications according to each database. Search was limited to the English language, and non-English articles were excluded.

### Study selection and data extraction

#### Selection process

We initially retrieved records from electronic databases and utilized EndNote software [18] to remove duplicate items. Thereafter, we uploaded the records and performed a two-step screening process using the Rayyan software [23]. Initially, two authors independently assessed the titles and abstracts of all included references to ascertain their relevance to our review. The next phase entailed evaluating the entire text of relevant publications for final eligibility. Discrepancies were handled by dialogue between the two authors, and a third author was consulted to see if the issue persisted.

### Data extraction

Two independent authors extracted data manually from studies on an online uniform data extraction Google sheet. Detailed information was carefully extracted from all eligible studies, including study ID (first author’s name + year of publication), design, sample size, included occlusion sites, characteristics of patients of included studies (including age, gender and comorbidities) and outcomes of interest.

### Assessment of risk of bias

Two authors performed a blinded evaluation of the quality of the selected studies. The risk for bias in the observational studies was assessed utilizing the Newcastle–Ottawa scale (NOS), which comprises three domains: selection process, comparability assessment, and assessment of reported outcomes. A third reviewer investigated the presence of any disagreements between the two authors.

### Statistical analysis and heterogeneity

Meta-analysis was performed using Review Manager software (RevMan v.5.4) [24] on the extracted outcome data that are present in at least two of the included studies. For dichotomous outcome data, the frequency of events and total number of patients were pooled as odds ratios (OR) with 95% confidence interval (CI). Regarding continuous outcomes, data were pooled as mean difference (MD) with 95% CI. The level of statistical significance was set to be p < 0.05. A random effect model (Inverse variance) was adopted rather than a fixed effect model, yielding a more conservative estimate of the pooled effect and generalizable results. To evaluate the presence and degree of heterogeneity, we used the chi-square and I-square tests, respectively [19]. We interpreted the I-square test results, which indicate the variation across studies due to heterogeneity rather than chance, as follows: 0–50% were considered insignificant, 50–70% were considered moderate, and more than 70% were considered substantial. Heterogeneity was considered significant if the alpha level for the chi-square test was below 0.1. A sensitivity analysis was conducted to assess the robustness of the results.

## Results

### Study selection

**Fig. 1:**
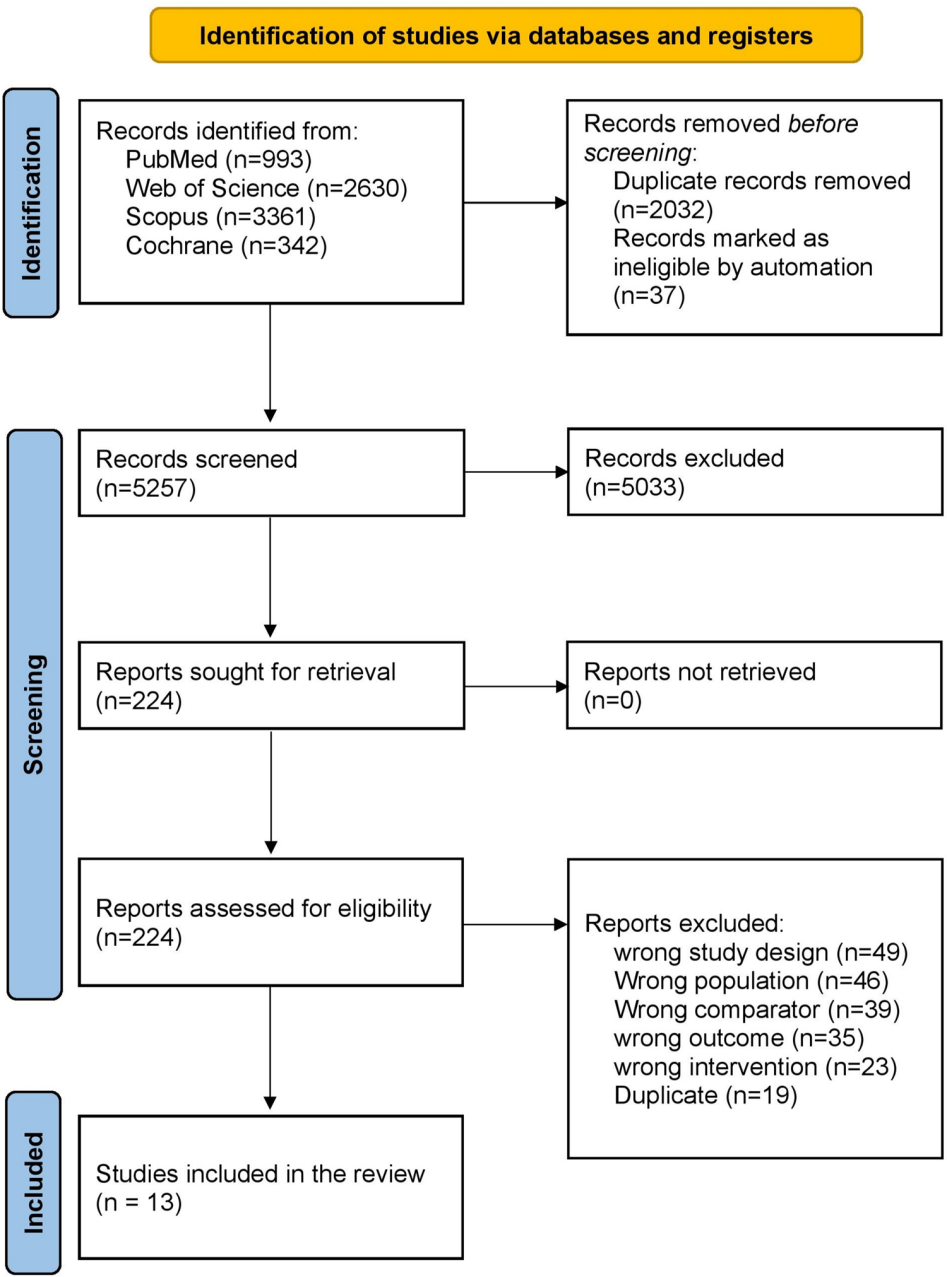
PRISMA flow diagram of the selection process

A comprehensive electronic literature search was conducted across PubMed, Cochrane, Web of Science, and Scopus databases, yielding a total of 7,326 records. After removing 2,032 duplicate entries and 37 entries identified by automation tools, 4,923 distinct studies were available for title and abstract screening. Initial screening of titles and abstracts resulted in the exclusion of 4,699 studies, leaving 224 articles for full-text assessment. Ultimately, 13 studies met all eligibility criteria and were included in this systematic review and meta-analysis. The detailed screening process is illustrated in the PRISMA flow diagram (Fig. 1).

### Study characteristics

The selection process resulted in 13 eligible studies comprising a total sample size of 6,198 patients [12–14, 16–25]. These included nine retrospective cohort studies, three prospective observational studies, and one post-hoc analysis of a randomized controlled trial (RCT). Studies were published between 2016 and 2025 and included adult patients undergoing mechanical thrombectomy (MT) for acute ischemic stroke. All included studies were multicentric, conducted across various regions, including Europe (Germany, Netherlands, Sweden, France, Austria), North America (USA, Canada), and Asia (Korea, China). The interventions primarily involved thrombectomy with or without intravenous thrombolysis. Detailed characteristics and baseline data of included studies are summarized in Tables 1 and 2.

**Table 1:** Summary of the included studies

Study ID	Study design	Country	Data source	Total number of participants	EVT Techniques	Primary outcome
Alves et al. 2016	Multi center observational, prospective study	Netherlands	MR CLEAN registry	1627	Catheterization only, DSA only, Mechanical thrombectomy	90-day functional outcome (mRS s)
Baik et al. 2016	Retrospective review	Korea	Retrospective review	98	Manual aspiration thrombectomy (MAT) using a Penumbra catheter	Overall rate of successful recanalization
Cohen et al. 2022	Retrospective analysis	France	Single regional thrombectomy capable university hospital	267	Mechanical thrombectomy	mRS 0–2 at 3 months
Flint et al. 2020	Retrospective analysis	United States	Retrospectively extracted from the electronic medical record and associated relational databases	327	Stentrievers and aspiration	Thrombus migration
Hansen et al. 2025	Retrospective analysis	Sweden	Two Swedish quality registries	1118	NR	90-day mRS 0–2
Kaesmacher et al. 2017	Retrospective analysis	Germany	A retrospective analysis of a prospective database	325	Aspiration and older devices (intra-arterial thrombolysis, Merci, stent retrievers)	Successful recanalization ≥ TICI-2b
Lee et al. 2020	Retrospective analysis	South Korea	Patient registry of endovascular reperfusion therapy (thrombectomy) at the Soonchunhyang University Bucheon Hospital	164	Stent retriever (Solitaire/Trevo) and/or aspiration (Penumbra) via balloon guide catheter	mRS 0–2 at 3 months
Ohara et al. 2020	Prospective observational study	Canada	Interrsect study	427	Stentriever and aspiration devices	mRS 0–2 at 3 months
Pikija et al. 2024	Retrospective analysis	Austria	Retrospectively analyzed data from two comprehensive Stroke centers in Austria Europe	463	Stentriever and aspiration devices	mRS 0–2 at 3 months
Rajah et al. 2020	Retrospective analysis	United States	A single-institution, prospectively maintained stroke database	80	Coaxial setup with guide catheter, stent retriever, intermediate large-bore aspiration catheter	Thrombus migration
Riegler et al. 2022	Observational study	Germany	The German Stroke Registry, a multicenter, prospective Registry assessing real-world outcomes	512	Stentriever and aspiration devices	mRS 0–2
Sporns et al. 2020	Retrospective analysis	Germany	Prospective cohort	163	Stent-retriever thrombectomy	Thrombus migration
Tan et al. 2023	Randomized controlled trial	China	Chinese Tertiary hospitals: A Multicentre randomised clinical Trial	627	NR	90-day mRS 0–2

**Table 2:** Baseline characteristics of the included studies

_Study ID_		_N (%)_	_Age, years, (mean ± SD)_	_Sex (Male)_ _N (%)_	_Baseline NIHSS, (mean ± SD)_	_occlusion site_					
						_ICA_	_ACA_	_MCA_	_PCA_	_Vertebra basilar_	_Tandem occlusion_
_Alves et al. 2016_	_Thrombus migration_	_302_	_69.5 (3.8)*_	_165 (55)_	_15.5 (1.4)_	_0 (0)_	_NA_	_302 (100)_	_0_	_0_	_0_
	_Thrombus resolution_	_39_	_70.75 (3.96)_	_32 (82)_	_14.5 (2.3)_	_N/A_	_NA_	_NA_			
	_Thrombus growth_	_87_	_68.75 (4.29)_	_47 (54)_	_15.5 (1.6)_	_47 (54)_	_NA_	_40(46)_			
	_Stable thrombus_	_921_	_70.25 (2.97)_	_476 (52)_	_16 (1.2)_	_215 (23)_	_NA_	_766 (84)_			
_Baik et al. 2016_	Migrated thrombus	_19_	_69.75 (2.44)_	_6 (31.6)_	_11.25 (1.35)_	_0_	_0_	_19 (100)_	_0_	_0_	_0_
	Non-migrated thrombus	_79_	_72 (3.7)_	_35 (44.3)_	_10.75 (1.86)_			_79 (100)_			
_Cohen et al. 2022_	Thrombus migration	_65_	_70 (11)_	_39 (60)_	_12.4 (6.9)_	_5 (7.7)_	_0_	_60 (92.3)_	_0_	_0_	_0_
	No thrombus migration	_202_	_70 (13)_	_104 (51)_	_15.1 (6.4)_	_27 (13.4)_		_175 (86.6)_			
_Flint et al. 2020_	EST only	_85_	_75.5 (3.3)_	_38 (44.7)_	_15.75 (3.07)_	_NR_					
	IV tPA + EST	_242_	_73 (3.6)_	_126 (52.1)_	_15.75 (1.97)_						
_Hansen et al. 2025_	Primary MCA MeVO	_819_	75.5 (2.2)	465 (57)	_10.5 (1.3)_	_NR_					
	Secondary MCA MeVO	_299_	74.25 (2.6)	152 (51)	_13.875 (1.833)_						
_Kaesmacher et al. 2017_	Thrombus migration	_97_	_70.5 (3.3)_	_45 (46.4)_	_14.75 (1.41)_	_NR_					
	No thrombus migration	_228_	_73 (3.6)_	_106 (46.3)_	_14 (1.1)_						
_Lee et al. 2020_	Thrombus migration	_32_	_70.8 (11)_	_12 (37.5)_	_15.1 (6.9)_	_NR_					
	No thrombus migration	_132_	_66.6 (13.5)_	_79 (59.8)_	_14.9 (6.1)_						
_Ohara et al. 2020_	Marked movement	_114_	_73 (3.54)_	_57 (50)_	_14.25 (1.96)_	_12 (11)_	_0_	_102 (89)_	_0_	_0_	_0_
	Mild to moderate movement	_116_	_72.5 (3.53)_	_47 (41)_	_14.75 (1.76)_	_27 (23)_		_89 (77)_			
_Pikija et al. 2024_	Stability	_357_	_75 (3.08)_	_144 (40)_	_16.5(1.37)_	_NR_					
	Migration	_78_	_75 (4.15)_	_40 (51)_	_16.25 (1.04)_						
_Rajah et al. 2020_	Yes tPA	_24_	_61.23 (2.86)_	_15 (33.33)_	_16.96 (1.6)_	_NR_					
	No tPA	_56_	_66.58 (1.39)_	_30 (66.67)_	_14.59 (0.87)_						
_Riegler et al. 2022_	Thrombus migration	_71_	_74.5 (3.79)_	_37 (52.1)_	_15.5 (2.1)_	NR					
	No thrombus migration	_441_	_75.25 (2.85)_	_191 (43.3)_	_14.75 (1.51)_						
_Sporns et al. 2020_	Clot migration	_36_	_69.075 (5.27)_	_18 (50)_	_14 (1.4)_	_NR_					
	No clot migration	_127_	_72.25 (3.68)_	_64 (50.3)_	_13 (1.2)_						
_Tan et al. 2023_	Thrombus migration	_71_	_67 (3.79)_	_45 (63.38)_	_14 (19.72)_	_NR_					
	No thrombus migration	_556_	_69.25 (2.45)_	_309 (55.58)_	_105 (18.88)_						

_Study ID_		_Preexisting medical conditions_								_Time metrics_		_IV Thrombolysis_ _N (%)_
		_Diabetes_	_HTN_	_Smoking_	_HF_	_AF_	_CAD/MI_	_Antiplatelet drug Use_	_Previous ischemic stroke/TIA_	_Onset/presentation to groin_	_Groin to recanalization_	
_Alves et al. 2016_	_Thrombus migration_	_50 (17)_	_149 (50)_	_NA_	_NA_	_53 (18)_	_44(15)_	_106 (36)_	_44 (15)_	_206.3 (81.1)_	_35.3 (11.1)_	_256 (85)_
	_Thrombus resolution_	_10 (26)_	_16 (41)_			_4 (11)_	_8 (38)_	_13 (33)_	_3 (8)_	_199.6 (60)_	_21(8.4)_	_33 (39)_
	_Thrombus growth_	_11 (13)_	_44 (51)_			_21 (24)_	_13 (87)_	_29 (33)_	_14 (16)_	_239.7 (87.4)_	_25 (7.5)_	_64 (87)_
	_Stable thrombus_	_151 (17)_	_468 (51)_			_221 (24)_	_141 (899)_	_296 (33)_	_152 (916)_	_213.3 (81.4)_	_25 (11.1)_	_679 (919)_
_Baik et al. 2016_	Migrated thrombus	_8 (42.9)_	_10 (57.1)_	_4 (28.6)_	_NA_	_4 (21.4)**_	_NR_	_NR_	_3 (14.3)_	_NR_	_13 (68.4)_	_NR_
	Non-migrated thrombus	_12 (15.2)_	_38 (48.1)_	_22 (17.8)_		_32 (40.5)**_			_14 (17.7)_		_37 (46.8)_	
_Cohen et al. 2022_	Thrombus migration	_12 (18.5)_	_40 (61.5)_	_9 (13.8)**_	_NR_	_13 (20)_	_NR_	_NR_	_NR_	_NR_	_NR_	_50 (77)_
	No thrombus migration	_34 (16.6)_	_122 (60.4)_	_35 (17.3)**_		_36 (17.8)_		_NR_				_99 (49)_
_Flint et al. 2020_	EST only	56 (65.90)	_29 (34.1)_	_N/A_	_NR_	25(29.40)	_21 (24.7)_	_13 (15.75)_	_20 (23.5)_	_115 (50.5)_	_NR_	_NR_
	IV tPA + EST	152 (63.20)	_71 (29.3)_	_N/A_		54(22.70)	_51 (21.1)_	_38 (15.75)_	_56 (23.1)_	_113.3 (44.6)_		
_Hansen et al. 2025_	Primary MCA MeVO	_489 (60)_	_169 (21)_	_88 (11)_	_NR_	_377 (46)_	_NR_	_NR_	_134 (16)_	_NR_	_NR_	_330 (40)_
	Secondary MCA MeVO	_182 (61)_	_48 (16)_	_20 (7)_		_125 (42)_		_NR_	_34 (11)_			_198 (66)_
_Kaesmacher et al. 2017_	Thrombus migration	21 (21.9)	74 (77.1)	_NR_	_NR_	21 (21.9)	_NR_	_NR_	_18 (18.8)_	_NR_	_62 (63.9)_	_NR_
	No thrombus migration	36 (15.9)	169 (74.8)	_NR_		36 (15.9)		_NR_	_41 (18.1)_		_148 (64.9)_	
_Lee et al. 2020_	Thrombus migration	10 (31.3)	22 (68.8)	_3 (9.4)_	_NR_	22 (68.8)	_NR_	_8 (25)_	_4 (12.5)_	_114.7 (103.3)_	_119.9 (53.8)_	_NR_
	No thrombus migration	39 (29.5)	85 (64.4)	_23 (17.4)_		66 (50)		_37 (28)_	_28 (21.2)_	_220.7 (364.3)_	_121.2 (70.6)_	
_Ohara et al. 2020_	Marked movement	14 (12)	71 (62)	_22 (20)_	_NR_	32 (28)	_NR_	_39 (34)_	_20 (18)_	_NR_	_NR_	_NR_
	Mild to moderate movement	20 (17)	82 (71)	_13 (11)_		35 (30)		_45 (39)_	_19 (17)_			
_Pikija et al. 2024_	Stability	37 (10)	194 (54)	_NR_	_NR_	134 (38)	_NR_	_58 (16)_	_42 (12)_	_186 (142,249)_	_76 (40,120)_	_NR_
	Migration	6 (7.7)	41 (53)			19 (24)		_16 (21)_	_17 (22)_	_234 (198,280)_	_105 (52,134)_	
_Rajah et al. 2020_	Yes tPA	6 (31.57)	21 (31.34)	_12 (32.43)_	_NR_	9 (31.03)	_NR_	_N/A_	_NR_	_NR_	_NR_	_NR_
	No tPA	13 (68.42)	46 (68.66)	_25 (67.57)_		20 (68.97)		_N/A_				
_Riegler et al. 2022_	Thrombus migration	11 (15.5)	54 (76.1)	_5 (7.6)_		38 (53.5)	_NR_	_21 (29.6)_	_NR_	_185 (74.3)_	_43.6 (25.5)_	_210 (47.6)_
	No thrombus migration	94 (21.3)	351 (79.6)	_61 (14.5)_		224 (50.8)		_129 (29.3)_		_171 (63.5)_	_37.6 (20.4)_	_53 (74.6)_
_Sporns et al. 2020_	Clot migration	7 (19.4)	27 (75)	_NR_	_NR_	N/A	_NR_	_N/A_	_NR_	_147 (74.8)_	_30 (13.1)_	_24 (69.4)_
	No clot migration	22 (17.3)	97 (76.4)			N/A		_N/A_		_138.3 (68.2)_	_32.6 (12.7)_	_108 (66.3)_
_Tan et al. 2023_	Thrombus migration	14 (19.72)	45 (63.38)	_NR_	_NR_	24 (33.8)	_NR_	_N/A_	_11 (15.49)_	_199 (64.3)_	_59.3 (35.5)_	_11 (15.49)_
	No thrombus migration	105 (18.88)	331 (59.53)			267 (48.02)		_N/A_	_75 (13.49)_	_205 (66.8)_	_67 (38.6)_	_75 (13.49)_

### Quality assessment

The risk of bias assessment was conducted using the Newcastle–Ottawa Scale (NOS), evaluating selection, comparability, and outcome domains. Most included studies demonstrated high methodological quality, scoring between 7 and 9 out of a possible 9 points. Specifically, six studies scored 9 points, indicating very high quality, while seven studies scored 8 points, indicating good quality. A detailed illustration of the risk of bias assessment is presented in Table 3.

**Table 3:** Risk-of-bias assessment results using the Newcastle–Ottawa Scale (NOS)

Study ID	Selection				Comparability	Outcome			Total score
	A	B	C	D	E	F	G	H	
Alves et al. 2019	*	*	*	*	*	*	*	*	8
Baik et al. 2016	*		*	*	*	*	*	*	7
Cohen et al. 2022	*	*	*	*	**	*	*	*	9
Flint et al. 2020	*	*	*	*	**	*	*	*	9
Hansen et al. 2025	*	*	*	*	**	*	*	*	9
Kaesmacher et al. 2017	*	*	*	*	*	*	*	*	8
Lee et al. 2020	*	*	*	*	*	*	*	*	8
Ohara et al. 2020	*	*	*	*	**	*	*	*	9
Pikijia et al. 2024	*	*	*	*	*	*	*	*	8
Rajah et al. 2020	*	*	*	*	**	*	*	*	9
Reigler et al. 2022	*	*	*	*	**	*	*	*	9
Sporns et al. 2020	*	*	*	*	*	*	*	*	8
Tan et al. 2023	*	*	*	*	*	*	*	*	8

A. Representativeness of the exposed cohort, B. Selection of the non-exposed cohort, C. Ascertainment of exposure, D. Demonstration that the outcome of interest was not present at the start of the study, E. Comparability of cohorts based on the design or analysis, F. Assessment of outcome, G. Was the follow-up long enough for outcomes to occur, H. Adequacy of follow-up of cohorts

### Clinical outcomes

#### 90-day favorable outcome (mRS 0–2)

**Fig. 2:**
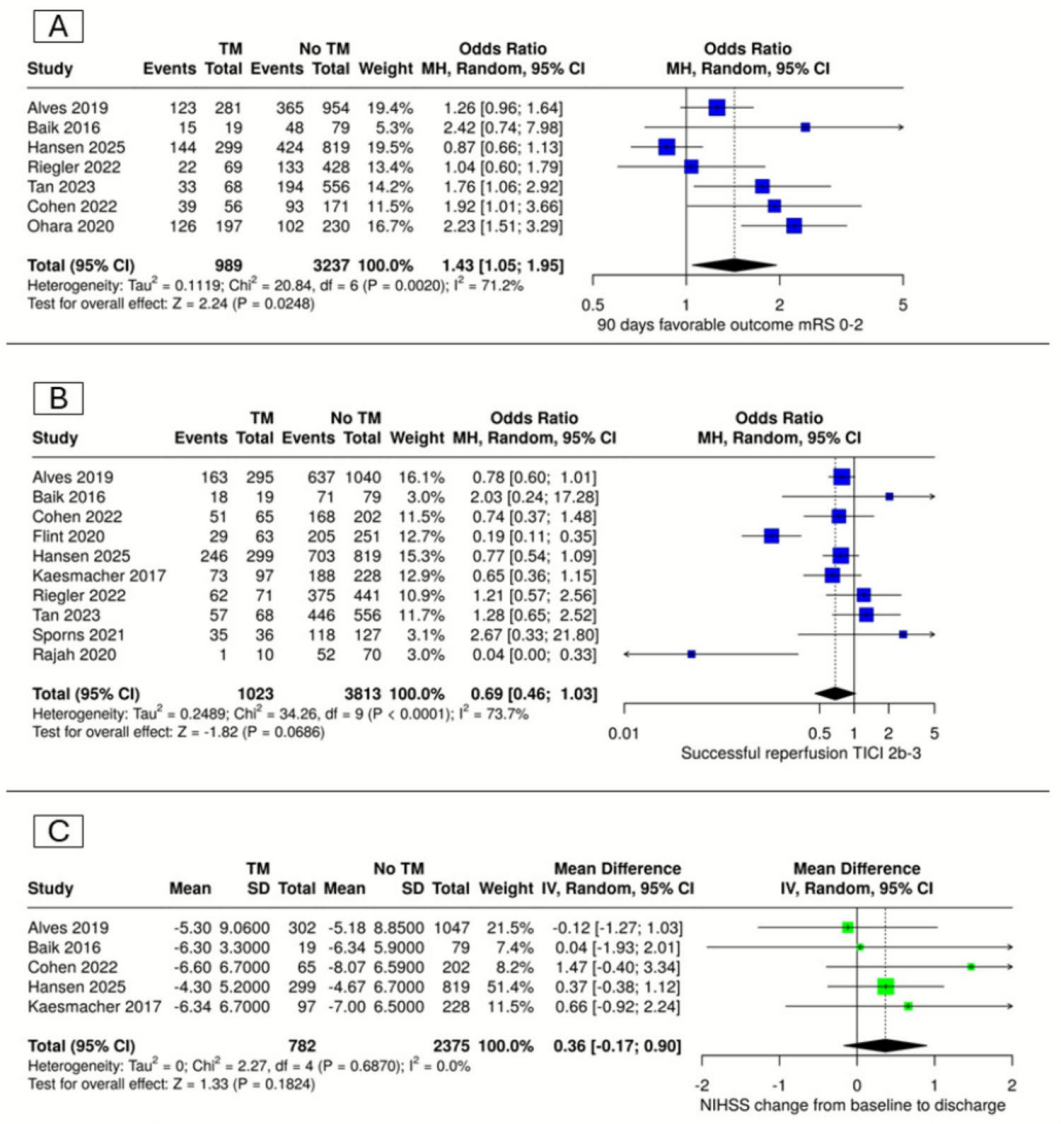
Forest plots of functional outcomes. (**A)** 90-Day favorable outcome (mRS 0–2), (**B**) successful reperfusion (TICI 2b-3), (**C**) NIHSS change from baseline to discharge

Seven studies assessed the impact of thrombus migration on favorable neurological outcomes at 90 days, defined as modified Rankin Scale (mRS) scores between 0 and 2. Meta-analysis demonstrated that thrombus migration was significantly associated with improved favorable outcomes compared to the non-migration group (OR = 1.43, 95% CI [1.05–1.95], P = 0.025). However, significant heterogeneity was observed (I^2^ = 71%; P = 0.002) (Fig. 2A).

#### Successful reperfusion (TICI 2b-3)

Meta-analysis of ten studies revealed no statistically significant difference in successful reperfusion rates (TICI 2b-3) between thrombus migration and non-migration groups (OR = 0.69, 95% CI [0.46–1.03], P = 0.07). Significant heterogeneity was identified (I^2^ = 73.7%; P < 0.0001) (Fig. 2B).

#### NIHSS change from baseline to discharge

Five studies assessed changes in NIHSS scores from baseline to discharge. There was no significant difference in neurological improvement between the thrombus migration and non-migration groups (MD = 0.36, 95% CI [-0.17–0.90], P = 0.18). No heterogeneity was detected (I^2^ = 0%; P = 0.69) (Fig. 2C).

#### 90-day mortality

**Fig. 3:**
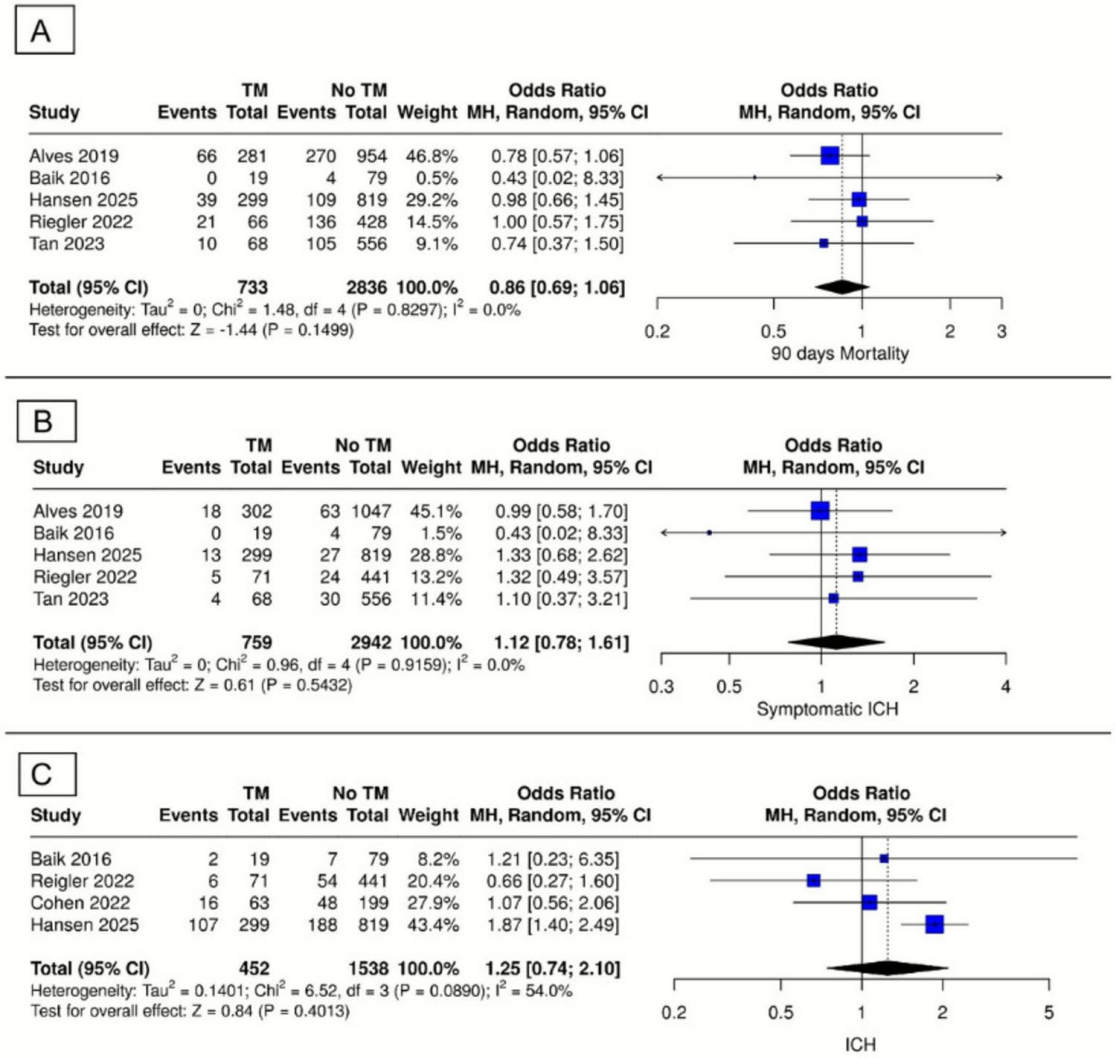
Forest plot of safety outcomes. (**A**) 90-Day mortality, (**B**) symptomatic intracranial haemorrhage (sICH), (**C**) any intracranial hemorrhage (ICH)

Analysis of five studies evaluating the impact of thrombus migration on 90-day mortality revealed no significant difference between thrombus migration and non-migration groups (OR = 0.86, 95% CI [0.69–1.06], P = 0.15). There was no heterogeneity among these studies (I^2^ = 0%; P = 0.83) (Fig. 3A).

#### Symptomatic intracranial haemorrhage (sICH)

Five studies evaluated the incidence of symptomatic intracranial haemorrhage. No significant difference between groups was observed (OR = 1.12, 95% CI [0.78–1.61], P = 0.54), with no heterogeneity (I^2^ = 0%; P = 0.92) (Fig. 3B).

#### Any intracranial haemorrhage (ICH)

Data from four studies indicated a statistically insignificant difference in ICH between the two groups (OR = 1.25, 95% CI [0.47–2.10], P = 0.48). moderate heterogeneity was observed (I^2^ = 54%; P = 0.1013) (Fig. 3C).

### Leave one out sensitivity analysis

We conducted sensitivity analyses to assess the single study effect on the robustness of the results (Supplementary file 2). For symptomatic ICH and mortality, odds ratios (ORs) remained close to 1.0 with low heterogeneity (I^2^ = 0%), suggesting consistent findings across studies. In contrast, favorable outcomes at 90 days showed more variability (I^2^ up to 76%), with statistical significance sensitive to certain study removals. 90 days Mortality and Mean change in NIHSS showed similar effect size upon removing any of the studies with homogenous results (I^2^ = 0). Successful reperfusion outcomes demonstrated moderate-to-high heterogeneity (I^2^ ≈ 70–77%), though effect sizes were relatively stable. Overall, the analysis suggests that no single study disproportionately influenced the meta-analysis conclusions, though some endpoints—particularly functional recovery and reperfusion—show notable heterogeneity that may reflect differences in patient populations or treatment protocols.

## Discussion

This meta-analysis suggests that the thrombus migration group was more likely to have favorable functional outcomes at 90 days. On the other hand, the migration and non-migration groups did not differ with respect to 90-day mortality, NIHSS score change from baseline, rates of successful reperfusion, or symptomatic intracranial haemorrhage.

Thrombus migration typically involves the movement of a thrombus from its original location in a proximal vessel to a more distal vessel, often involving fragmentation. Many studies have implicated thrombus migration in less successful revascularization due to the technical challenges it presents. Migration displaces the occlusion to smaller branches that are often less accessible to endovascular devices. However, in our meta-analysis, there was no statistically significant difference between the thrombus migration and non-migration groups in terms of successful revascularization, although there was a trend towards less successful revascularization in the thrombus migration group. Notably, there was significant heterogeneity, suggesting that many factors, including technical skill, the time interval between imaging and intervention, and specific migration patterns, may affect the rate of complete recanalization and successful revascularization [12, 15, 24].

Incomplete recanalization following EVT in patients with thrombus migration is commonly attributable to persistent distal emboli beyond the reach of conventional thrombectomy devices. In selected patients, adjunctive therapies such as additional distal thrombectomy manoeuvres, angioplasty, or post-thrombectomy intra-arterial thrombolysis may be considered to address these residual occlusions. Of note, post-thrombectomy intra-arterial thrombolysis is showing promising results even after successful recanalization and should be considered in patients with thrombus migration [26].

Despite offering no apparent advantage for achieving revascularization, the effects of thrombus migration on functional outcomes are more nuanced. Its association with better or worse clinical outcomes necessitates an understanding of how the thrombus evolves during migration. Migration of a thrombus to a more distal vessel relieves the blockage in the proximal vessel, which generally supplies a larger intracranial territory and may thereby restore blood flow to areas originally at risk. The INTERRSeCT study demonstrated that marked alteplase-induced thrombus migration in patients with proximal M1 or ICA occlusions resulted in favorable outcomes in 52% of cases, compared to only 27% in those with limited thrombus movement [18]. Similarly, analysis from the MR CLEAN registry found that, in patients with M1 occlusion, those with thrombus migration were nearly 50% more likely to achieve better functional outcomes at 90 days [14]. These findings support the results of our meta-analysis, suggesting that migration to more distal vessels may reduce intracranial damage by salvaging proximal territories that would have otherwise been at high risk of ischemia. There is also a suggestion in the current literature of a dose–response relationship, in which more distal thrombus migration correlates with a reduced risk of significant damage to brain tissue [18]. Notably, it should be emphasized that distal thrombi are not inconsequential. Rather, this suggest that thrombus migration may reflect partial reperfusion with residual distal hypoperfusion, a state in which adjunctive therapies such as post-EVT intra-arterial thrombolysis may confer additional benefit. Furthermore, in our analysis, we compare thrombus migration vs. non-thrombus migration, which does not mean that the thrombus migration group does not receive any adjunct therapy. Instead, the treatment plan can be changed depending on thrombus migration, and this has nothing to do with our analysis, as we are interested in the overall outcomes after receiving the best treatment.

Although thrombus migration results in the distal relocation of the occlusion and potentially preserves the ischemic penumbra, our meta-analysis did not demonstrate an associated reduction in 90-day mortality. This highlights that mortality is governed by more complex clinical and systemic determinants, including baseline clinical severity and systemic complications, that extend beyond the location of the thrombus. These findings underscore that thrombus migration may be more relevant for predicting functional recovery than for overall survival and highlight the importance of including both functional and mortality endpoints in future studies.

### Clinical implication

The clinical implication of this meta-analysis is that thrombus migration often occurs with IV thrombolysis using alteplase and may not be a wholly harmful phenomenon. While it may complicate subsequent endovascular intervention and reduce the likelihood of complete recanalization, it has been shown to improve clinical outcomes in patients who have it.

### Recommendations:

Future research should focus on potential causes of thrombus migration and consider IV thrombolytics as a possible contributing factor. Furthermore, they should investigate whether the clinical outcomes differ depending on receiving IV thrombolytics or not. More prospective studies focusing on the effects of thrombus migration will be invaluable as the current evidence is based mainly on retrospective studies. In addition, as time is a crucial determinant of the outcome, investigating the outcomes of thrombus migration based on time would be highly beneficial. Although knowing exactly when thrombus migration occurs is very challenging, using CT or MRI to DSA or even onset/presentation to groin intervals as rough estimates can be beneficial. Defining a cutoff point for early vs. late thrombus migration based on these intervals would help to see whether time affects the effect of thrombus migration or not.

### Strengths and limitations:

The strengths of this meta-analysis include its incorporation of many studies from multiple countries and a sizable pooled sample of 6,198 patients. However, several limitations should be noted. Not all studies assessed each of the primary outcomes of interest, and significant heterogeneity, particularly regarding 90-day functional outcomes and successful reperfusion rates, limited the ability to draw definitive conclusions about their association with thrombus migration.

## Conclusion

This systematic review and meta-analysis demonstrates that thrombus migration in patients with acute ischemic stroke undergoing endovascular treatment is associated with improved functional outcomes at 90 days, without a corresponding effect on mortality, hemorrhagic complications, or overall rates of successful reperfusion. These findings suggest that thrombus migration is not always unfavorable and may, in some cases, confer clinical benefit by relieving proximal arterial occlusion and preserving larger volumes of at-risk brain tissue. However, thrombus migration showed a trend towards unsuccessful recanalization due to residual distal emboli, underscoring the importance of adjunctive treatments to optimize distal reperfusion. Collectively, our results highlight the complex and dual nature of thrombus migration, functioning both as a marker of partial reperfusion and a potential contributor to favorable neurological recovery. Future prospective studies are needed to clarify the mechanisms, timing, and clinical modifiers of thrombus migration, as well as to define optimal treatment strategies in this setting, particularly in relation to bridging thrombolysis and adjunctive post-thrombectomy therapies.

## Supplementary Information

Below is the link to the electronic supplementary material.

Supplementary file 1.

Supplementary file 2.

## Data Availability

All data analyzed were derived from published articles included in this systematic review. The data extraction sheet and analytic files are available from the corresponding author upon reasonable request.
