# Supplementary material for: Effects of thrombus migration on endovascular treatment outcomes in patients with ischemic stroke: a systematic review and meta-analysis
Source: J Neurol. 2026 Mar 9;273(3):189. doi: 10.1007/s00415-025-13609-9 (PMC12971846; doi:10.1007/s00415-025-13609-9)
Supplement: Supplementary file 1 [file 415_2025_13609_MOESM1_ESM.pdf]

# Effects Of Thrombus Migration On Endovascular Treatment Outcomes In Patients With Ischemic Stroke: A Systematic Review And Meta-Analysis

Hesham Kelani[1⁋], Mohamed A. Elzayat[2⁋*], Abdelrahman M. Elettreby[2], Hend Heikal[3], Maria Farag[3], Emily Wen Jing Shuai[3], Bethany Jordyn Thach[3], Hamza Khelifa[4], Emily Carrieri[3], Gabriela Martin Gonzalez[3], Vaughn Sherman[5], Ahmed Abd Elazim[6], Diana Greene-Chandos[7], Volodymyr Vulkanov[8], Moshe Mizrahi[1], Lisa R. Merlin[1,9], David Rosenbaum-Halevi[1], Priyank Khandelwal[9].

1. Department of Neurology, SUNY Downstate Health Sciences University at One Brooklyn Health, Brooklyn, NY.
2. Faculty of Medicine, Mansoura University, Mansoura, Egypt
3. College of Medicine, SUNY Downstate Health Sciences University, Brooklyn, NY
4. Faculty of Medicine. University of Oran 1 Ahmed Ben Bella. Oran. Algeria
5. Touro College of Osteopathic Medicine (TOUROCOM) - Harlem, NY Campus.
6. Department of Neurology, Sanford USD Medical Center, Sioux Falls, SD.
7. Department of Neurology, School of Medicine, University of Saint Louis, Saint Louis, MO.
8. Department of Neurology, Rutgers, New Jersey School of Medicine, Newark, NJ.
9. Departments of Neurology, Physiology and Pharmacology, SUNY Downstate Health Sciences University, Brooklyn, NY.

⁋ Hesham Kelani and Mohamed A. Elzayat contributed equally to this work

**Correspondence to:** Volodymyr Vulkanov; vv263@njms.rutgers.edu and Abdelrahman M. Elettreby; Am731784@gamil.com

## *Cochrane:*

#1 "cerebrovascular disorders" OR "basal ganglia cerebrovascular disease" OR "brain ischemia" OR "carotid artery diseases" OR "carotid artery thrombosis" OR "carotid artery, internal, dissection" OR "intracranial arterial diseases" OR "cerebral arterial diseases"

#2 "anterior cerebral artery infarction" OR "middle cerebral artery infarction" OR "posterior cerebral artery infarction" OR "infarction, anterior cerebral artery" OR "infarction, middle cerebral artery" OR "infarction, posterior cerebral artery"

#3 "Lacunar Infarct" OR "Lacunar Infarction" OR "intracranial embolism" OR "intracranial thrombosis" OR "brain infarction" OR "vertebral artery dissection"

#4 ((brain OR cerebr* OR cerebell* OR vertebrobasil* OR hemispher* OR intracran* OR intracerebral OR infratentorial OR supratentorial OR middle cerebr* OR anterior circulation) near/5 (Ischemi* OR Ischaemi* OR infarct* OR thrombo* OR emboli* OR occlus* OR hypoxi*))

#5 ((Ischemi* OR Ischaemi*) near/6 (stroke* OR apoplex* OR cerebral vasc* OR cerebrovasc* OR cva OR attack*))

#6 "cerebral sinus thrombosis" OR "cerebral venous sinus thrombosis" OR "CVST" OR "CVT"

#7 #1 OR #2 OR #3 OR #4 OR #5 OR #6

#8 (Thromb* OR Clot* OR Embol*) near/2 (migration OR movement OR displacement OR shift OR mobilization OR mobilisation OR mobility OR dynamics OR fragmentation OR dislodgement)

#9 (distal OR mobile OR migrated OR migrating OR displaced OR secondary OR delayed OR dynamic OR shifting) near/2 (Thromb* OR Clot* OR Embol*)

#10 #8 OR #9

#11 #7 AND #10

## PubMed:

#1 "Ischemic Stroke"[Mesh] OR stroke OR "acute stroke*" OR "ischemic stroke" OR "acute ischemic stroke" OR " Embolic Stroke" OR " Thrombotic Stroke" OR "cerebrovascular Accident*" OR "cerebral vascular accident*" OR "Brain Vascular Accident*" OR "cerebrovascular disorders" OR "basal ganglia cerebrovascular disease" OR "thrombotic events" OR "thromboses"

#2 "acute cerebral ischemia" OR "cerebral ischemia" OR "brain ischemia" OR "cerebral infarction" OR "brain infarction" OR "Lacunar Infarct" OR "Lacunar Infarction" OR "intracranial embolism" OR "intracranial thrombosis" OR "vertebral artery dissection"

#3 "carotid artery diseases" OR "carotid artery thrombosis" OR "carotid artery, internal, dissection" OR "intracranial arterial diseases" OR "cerebral arterial diseases" OR "cerebrovascular apoplex*"

#4 "cerebral arterial occlusion" OR "cerebral artery occlusion" OR "anterior cerebral artery infarction" OR "middle cerebral artery infarction" OR "posterior cerebral artery infarction" OR "infarction, anterior cerebral artery" OR "infarction, middle cerebral artery" OR "infarction, posterior cerebral artery" OR "cerebral sinus thrombosis" OR "cerebral venous sinus thrombosis" OR "CVST" OR "CVT"

#5 #1 OR #2 OR #3 OR #4

#6 "thrombus migration"[Title/Abstract:~2] OR "thrombi migration"[Title/Abstract:~2] OR "thrombotic migration"[Title/Abstract:~2] OR "thrombus movement"[Title/Abstract:~2] OR "thrombi movement"[Title/Abstract:~2] OR "thrombotic movement"[Title/Abstract:~2] OR "thrombus displacement"[Title/Abstract:~2] OR "thrombi displacement"[Title/Abstract:~2] OR "thrombotic displacement"[Title/Abstract:~2] OR "thrombus shift"[Title/Abstract:~2] OR "thrombi shift"[Title/Abstract:~2] OR "thrombotic shift"[Title/Abstract:~2] OR "thrombus mobilization"[Title/Abstract:~2] OR "thrombi mobilization"[Title/Abstract:~2] OR "thrombotic mobilization"[Title/Abstract:~2] OR "thrombus mobilisation"[Title/Abstract:~2] OR "thrombi mobilisation"[Title/Abstract:~2] OR "thrombotic mobilisation"[Title/Abstract:~2] OR "thrombus mobility"[Title/Abstract:~2] OR "thrombi mobility"[Title/Abstract:~2] OR "thrombotic mobility"[Title/Abstract:~2] OR "thrombus dynamics"[Title/Abstract:~2] OR "thrombi dynamics"[Title/Abstract:~2] OR "thrombotic dynamics"[Title/Abstract:~2] OR "thrombus fragmentation"[Title/Abstract:~2] OR "thrombi fragmentation"[Title/Abstract:~2] OR "thrombotic fragmentation"[Title/Abstract:~2] OR "thrombus dislodgement"[Title/Abstract:~2] OR "thrombi dislodgement"[Title/Abstract:~2] OR "thrombotic dislodgement"[Title/Abstract:~2]

#7 "clot migration"[Title/Abstract:~2] OR "clot movement"[Title/Abstract:~2] OR "clot displacement"[Title/Abstract:~2] OR "clot shift"[Title/Abstract:~2] OR "clot mobilization"[Title/Abstract:~2] OR "clot mobilisation"[Title/Abstract:~2] OR "clot mobility"[Title/Abstract:~2] OR "clot dynamics"[Title/Abstract:~2] OR "clot fragmentation"[Title/Abstract:~2] OR "clot dislodgement"[Title/Abstract:~2] OR "clots migration"[Title/Abstract:~2] OR "clots movement"[Title/Abstract:~2] OR "clots displacement"[Title/Abstract:~2] OR "clots shift"[Title/Abstract:~2] OR "clots mobilization"[Title/Abstract:~2] OR "clots mobilisation"[Title/Abstract:~2] OR "clots mobility"[Title/Abstract:~2] OR "clots dynamics"[Title/Abstract:~2] OR "clots fragmentation"[Title/Abstract:~2] OR "clots dislodgement"[Title/Abstract:~2]

#8 "emboli migration"[Title/Abstract:~2] OR "emboli movement"[Title/Abstract:~2] OR "emboli displacement"[Title/Abstract:~2] OR "emboli shift"[Title/Abstract:~2] OR "emboli

mobilization"[Title/Abstract:~2] OR "emboli mobilisation"[Title/Abstract:~2] OR "emboli mobility"[Title/Abstract:~2] OR "emboli dynamics"[Title/Abstract:~2] OR "emboli fragmentation"[Title/Abstract:~2] OR "emboli dislodgement"[Title/Abstract:~2] OR "embolus migration"[Title/Abstract:~2] OR "embolus movement"[Title/Abstract:~2] OR "embolus displacement"[Title/Abstract:~2] OR "embolus shift"[Title/Abstract:~2] OR "embolus mobilization"[Title/Abstract:~2] OR "embolus mobilisation"[Title/Abstract:~2] OR "embolus mobility"[Title/Abstract:~2] OR "embolus dynamics"[Title/Abstract:~2] OR "embolus fragmentation"[Title/Abstract:~2] OR "embolus dislodgement"[Title/Abstract:~2]

#9 "embolic migration"[Title/Abstract:~2] OR "embolic movement"[Title/Abstract:~2] OR "embolic displacement"[Title/Abstract:~2] OR "embolic shift"[Title/Abstract:~2] OR "embolic mobilization"[Title/Abstract:~2] OR "embolic mobilisation"[Title/Abstract:~2] OR "embolic mobility"[Title/Abstract:~2] OR "embolic dynamics"[Title/Abstract:~2] OR "embolic fragmentation"[Title/Abstract:~2] OR "embolic dislodgement"[Title/Abstract:~2] OR "thromboembolic migration"[Title/Abstract:~2] OR "thromboembolic movement"[Title/Abstract:~2] OR "thromboembolic displacement"[Title/Abstract:~2] OR "thromboembolic shift"[Title/Abstract:~2] OR "thromboembolic mobilization"[Title/Abstract:~2] OR "thromboembolic mobilisation"[Title/Abstract:~2] OR "thromboembolic mobility"[Title/Abstract:~2] OR "thromboembolic dynamics"[Title/Abstract:~2] OR "thromboembolic fragmentation"[Title/Abstract:~2] OR "thromboembolic dislodgement"[Title/Abstract:~2]

#10 "distal thrombus"[Title/Abstract:~2] OR "distal thrombi"[Title/Abstract:~2] OR "distal embolus"[Title/Abstract:~2] OR "distal emboli"[Title/Abstract:~2] OR "distal clot"[Title/Abstract:~2] OR "distal clots"[Title/Abstract:~2] OR "mobile thrombus"[Title/Abstract:~2] OR "mobile thrombi"[Title/Abstract:~2] OR "mobile embolus"[Title/Abstract:~2] OR "mobile emboli"[Title/Abstract:~2] OR "mobile clot"[Title/Abstract:~2] OR "mobile clots"[Title/Abstract:~2]

#11 "migrated thrombus"[Title/Abstract:~2] OR "migrated thrombi"[Title/Abstract:~2] OR "migrated embolus"[Title/Abstract:~2] OR "migrated emboli"[Title/Abstract:~2] OR "migrated clot"[Title/Abstract:~2] OR "migrated clots"[Title/Abstract:~2] OR "migrating thrombus"[Title/Abstract:~2] OR "migrating thrombi"[Title/Abstract:~2] OR "migrating embolus"[Title/Abstract:~2] OR "migrating emboli"[Title/Abstract:~2] OR "migrating clot"[Title/Abstract:~2] OR "migrating clots"[Title/Abstract:~2] OR "displaced thrombus"[Title/Abstract:~2] OR "displaced thrombi"[Title/Abstract:~2] OR "displaced embolus"[Title/Abstract:~2] OR "displaced emboli"[Title/Abstract:~2] OR "displaced clot"[Title/Abstract:~2] OR "displaced clots"[Title/Abstract:~2]

#12 "secondary thrombus"[Title/Abstract:~2] OR "secondary thrombi"[Title/Abstract:~2] OR "secondary embolus"[Title/Abstract:~2] OR "secondary emboli"[Title/Abstract:~2] OR "secondary clot"[Title/Abstract:~2] OR "secondary clots"[Title/Abstract:~2] OR "delayed thrombus"[Title/Abstract:~2] OR "delayed thrombi"[Title/Abstract:~2] OR "delayed embolus"[Title/Abstract:~2] OR "delayed emboli"[Title/Abstract:~2] OR "delayed clot"[Title/Abstract:~2] OR "delayed clots"[Title/Abstract:~2]

#13 "dynamic thrombus"[Title/Abstract:~2] OR "dynamic thrombi"[Title/Abstract:~2] OR "dynamic embolus"[Title/Abstract:~2] OR "dynamic emboli"[Title/Abstract:~2] OR "dynamic clot"[Title/Abstract:~2] OR "dynamic clots"[Title/Abstract:~2] OR "shifting thrombus"[Title/Abstract:~2] OR "shifting thrombi"[Title/Abstract:~2] OR "shifting embolus"[Title/Abstract:~2] OR "shifting emboli"[Title/Abstract:~2] OR "shifting clot"[Title/Abstract:~2] OR "shifting clots"[Title/Abstract:~2]

#14 #6 OR #7 OR #8 OR #9 OR #10 OR #11 OR #12 OR #13

#15 #5 AND #14

**RESULTS: 993**

## WOS:

#1 ALL=("cerebrovascular disorders" OR "basal ganglia cerebrovascular disease" OR "carotid artery thrombosis" OR "carotid artery, internal, dissection" OR "cerebral arterial diseases" OR "cerebral sinus thrombosis" OR "cerebral venous sinus thrombosis" OR "CVST" OR "CVT" OR "anterior cerebral artery infarction" OR "middle cerebral artery infarction" OR "posterior cerebral artery infarction" OR "Lacunar Infarction" OR "intracranial embolism" OR "intracranial thrombosis" OR "brain infarction" OR "vertebral artery dissection" OR "brain ischemia")
#2 TS=((("Ischemi*" OR "Ischaemi*") near/6 ("stroke*" OR "apoplex*" OR "cerebral vasc*" OR "cerebrovasc*" OR cva OR "attack*")))
#3 TS=(((brain OR "cerebr*" OR "cerebell*" OR "vertebrobasil*" OR "hemispher*" OR "intracran*" OR intracerebral OR infratentorial OR supratentorial OR "middle cerebr*" OR "anterior circulation") near/5 ("Ischemi*" OR "Ischaemi*" OR "infarct*" OR "thrombo*" OR "emboli*" OR "occlus*" OR "hypoxi*")))
#4 #1 OR #2 OR #3

#5 TS=((Thromb* OR Clot* OR Embol*) near/2 (migration OR movement OR displacement OR shift OR mobilization OR mobilisation OR mobility OR dynamics OR fragmentation OR dislodgement))

#6 TS=((distal OR mobile OR migrated OR migrating OR displaced OR secondary OR delayed OR dynamic OR shifting) near/2 (Thromb* OR Clot* OR Embol*))

#7 #5 OR #6
#8 #4 AND #7
**RESULTS:2630**

## SCOPUS:

( TITLE-ABS-KEY ( "cerebrovascular disorders" OR "basal ganglia cerebrovascular disease" OR "brain ischemia" OR "carotid artery diseases" OR "carotid artery thrombosis" OR "carotid artery, internal, dissection" OR "intracranial arterial diseases" OR "cerebral arterial diseases" ) OR TITLE-ABS-KEY ( "anterior cerebral artery infarction" OR "middle cerebral artery infarction" OR "posterior cerebral artery infarction" OR "infarction, anterior cerebral artery" OR "infarction, middle cerebral artery" OR "infarction, posterior cerebral artery" ) OR TITLE-ABS-KEY ( "Lacunar Infarct" OR "Lacunar Infarction" OR "intracranial embolism" OR "intracranial thrombosis" OR "brain infarction" OR "vertebral artery dissection" ) OR TITLE-ABS-KEY ( ( ( brain OR cerebr* OR cerebell* OR vertebrobasil* OR hemispher* OR intracran* OR intracerebral OR infratentorial OR supratentorial OR "middle cerebr*" OR "anterior circulation" ) W/5 ( ischemi* OR ischaemi* OR infarct* OR thrombo* OR emboli* OR occlus* OR hypoxi* ) ) ) OR TITLE-ABS-KEY ( ( ( ischemi* OR ischaemi* ) W/6 ( stroke* OR apoplex* OR "cerebral vasc*" OR cerebrovasc* OR cva OR attack* ) ) ) OR TITLE-ABS-KEY ( "cerebral sinus thrombosis" OR "cerebral venous sinus thrombosis" OR "CVST" OR "CVT" ) )

**AND**

( TITLE-ABS-KEY ( ( thromb* OR clot* OR embol* ) W/2 ( migration OR movement OR displacement OR shift OR mobilization OR mobilisation OR mobility OR dynamics OR fragmentation OR dislodgement ) ) OR TITLE-ABS-KEY ( ( distal OR mobile OR migrated OR migrating OR displaced OR secondary OR delayed OR dynamic OR shifting ) W/2 ( thromb* OR clot* OR embol* ) ) )

**RESULTS: 3,361**

---

**TOTAL: 7326**
