# Supplementary material for: Effects of thrombus migration on endovascular treatment outcomes in patients with ischemic stroke: a systematic review and meta-analysis
Source: J Neurol. 2026 Mar 9;273(3):189. doi: 10.1007/s00415-025-13609-9 (PMC12971846; doi:10.1007/s00415-025-13609-9)
Supplement: Supplementary file 2 [file 415_2025_13609_MOESM2_ESM.pdf]

Effects Of Thrombus Migration On Endovascular Treatment Outcomes In Patients With Ischemic Stroke: A Systematic Review And Meta-Analysis

Hesham Kelani[1⁋], Mohamed A. Elzayat[2 ⁋*], Abdelrahman M. Elettreby[2], Hend Heikal[3], Maria Farag[3], Emily Wen Jing Shuai[3], Bethany Jordyn Thach[3], Hamza Khelifa[4], Emily Carrieri[3], Gabriela Martin Gonzalez[3], Vaughn Sherman[5], Ahmed Abd Elazim[6], Diana Greene-Chandos[7], Volodymyr Vulkanov[8], Moshe Mizrahi[1], Lisa R. Merlin[1,9], David Rosenbaum-Halevi[1], Priyank Khandelwal[9].

1. Department of Neurology, SUNY Downstate Health Sciences University at One Brooklyn Health, Brooklyn, NY.
2. Faculty of Medicine, Mansoura University, Mansoura, Egypt
3. College of Medicine, SUNY Downstate Health Sciences University, Brooklyn, NY
4. Faculty of Medicine. University of Oran 1 Ahmed Ben Bella. Oran. Algeria
5. Touro College of Osteopathic Medicine (TOUROCOM) - Harlem, NY Campus.
6. Department of Neurology, Sanford USD Medical Center, Sioux Falls, SD.
7. Department of Neurology, School of Medicine, University of Saint Louis, Saint Louis, MO.
8. Department of Neurology, Rutgers, New Jersey School of Medicine, Newark, NJ.
9. Departments of Neurology, Physiology and Pharmacology, SUNY Downstate Health Sciences University, Brooklyn, NY.

⁋ Hesham Kelani and Mohamed A. Elzayat contributed equally to this work

Correspondence to: Volodymyr Vulkanov; vv263@njms.rutgers.edu and Abdelrahman M. Elettreby; Am731784@gamil.com

TableS1: Leave-one-out analysis to detect the single study effect.

Study removed	No. of Participants	No. of trials	Quantitative data synthesis			Heterogeneity analysis	
			OR	95% CI	P-value	P-value	I^2(%)
**Symptomatic ICH**							
Alves 2019	2352	4	1.238 [0.759 - 2.019]		0.39	0.89	0
Baik 2016	3603	4	1.135 [0.788 - 1.636]		0.50	0.91	0
Hansen 2025	2583	4	1.042 [0.678 - 1.602]		0.85	0.89	0
Riegler 2022	3189	4	1.092 [0.74 - 1.611]		0.66	0.84	0
Tan 2023	3077	4	1.122 [0.763 - 1.649]		0.56	0.81	0
**90 days favorable outcome mRS 0-2**							
Alves 2019	2991	6	1.5 [0.99 - 2.27]		0.05	0.0009	76
Baik 2016	4129	6	1.39 [1.01 - 1.92]		0.046	0.0014	75
Hansen 2025	3108	6	1.59 [1.22 - 2.08]		0.00074	0.11	44
Riegler 2022	3729	6	1.51 [1.06 - 2.15]		0.02	0.0011	75
Tan 2023	3602	6	1.39 [0.98 - 1.96]		0.07	0.0018	74
Cohen 2022	3999	6	1.38 [0.98 - 1.93]		0.06	0.0018	74
Ohara 2020	3799	6	1.27 [0.96 - 1.69]		0.09	0.04	57

**90 days Mortality**						
Alves 2019	2334	4	0.93 [0.69 - 1.25]	0.63	0.85	0
Baik 2016	3471	4	0.86 [0.69 - 1.06]	0.16	0.74	0
Hansen 2025	2451	4	0.81 [0.63 - 1.04]	0.10	0.83	0
Riegler 2022	3075	4	0.83 [0.66 - 1.05]	0.12	0.77	0
Tan 2023	2945	4	0.87 [0.69 - 1.08]	0.21	0.73	0
**Successful reperfusion TICI 2b-3**						
Alves 2019	3501	9	0.67 [0.39 - 1.14]	0.14	< 0.0001	76
Baik 2016	4738	9	0.66 [0.44 - 1.00]	0.05	< 0.0001	76
Cohen 2022	4569	9	0.68 [0.43 - 1.07]	0.09	< 0.0001	77
Flint 2020	4522	9	0.82 [0.62 - 1.09]	0.17	0.0971	41
Hansen 2025	3718	9	0.67 [0.40 - 1.12]	0.13	< 0.0001	77
Kaesmacher 2017	4511	9	0.69 [0.43 - 1.10]	0.12	< 0.0001	77
Riegler 2022	4324	9	0.64 [0.41 - 0.99]	0.04	< 0.0001	75
Tan 2023	4212	9	0.63 [0.41 - 0.98]	0.04	< 0.0001	74
Sporns 2021	4673	9	0.66 [0.44 - 0.99]	0.05	< 0.0001	76
Rajah 2020	4756	9	0.74 [0.51 - 1.08]	0.12	0.0007	70

**Hemorrhage events**						
Baik 2016	1380	2	1.53 [0.91 - 2.59]	0.11	0.13	57
Hansen 2025	360	2	1.13 [0.63 - 2.01]	0.67	0.75	0
Cohen 2022	1216	2	1.84 [1.39 - 2.44]	<0.0001	0.62	0
**Study removed**	**No. of Participants**	**No. of trials**	**Quantitative data synthesis**		**Heterogeneity analysis**	
			**MD 95% CI**	**P-value**	**P-value**	**I^2(%)**
**Mean change NIHSS**						
Alves 2019	1808	4	0.50 [-0.11 - 1.10]	0.11	0.70	0
Baik 2016	3059	4	0.39 [-0.17 - 0.95]	0.17	0.54	0
Cohen 2022	2890	4	0.26 [-0.29 - 0.82]	0.35	0.85	0
Hansen 2025	2039	4	0.36 [-0.41 - 1.13]	0.36	0.52	0
Kaesmacher 2017	3157	4	0.36 [-0.17 - 0.9]	0.18	0.69	0
